# Donor oocyte cycle characteristics and outcomes: factors potentially linked with successful endings

**DOI:** 10.5935/1518-0557.20220046

**Published:** 2023

**Authors:** Fernanda K. Robin, Gabriella M. Andrade, Adriana Bos-Mikich, Nilo Frantz, Denise A. A. Zocche, Sandra M. C. Leal

**Affiliations:** 1 Nilo Frantz Reproductive Medicine, Porto Alegre, RS, Brazil; 2 Rio Grande do Sul Federal University, Porto Alegre, RS, Brazil; 3 Santa Catarina State University, Nursing Department, SC, Brazil; 4 Vale do Rio dos Sinos University, Nursing Department, Porto Alegre, RS, Brazil

**Keywords:** donor oocyte, pregnancy, live birth rates, diminished ovarian reserve

## Abstract

**Objective:**

The use of donor oocytes in assisted reproduction has seen a significant rise worldwide in the last two decades. Postponement of motherhood and premature ovarian insufficiency are the main reasons for the increase in the number of in vitro fertilization cycles with donor oocytes. The present study aims to characterize donor oocyte cycles to analyze factors that may have an effect on live births and clinical pregnancy outcomes.

**Methods:**

Data were obtained from a single Assisted Reproduction Center in southern Brazil. Recipient demographics (n=148 patients) and cycle characteristics (n=213 cycles; 50 patients did more than one IVF attempt) were analyzed. Statistical analysis was performed using chi-squared and t-test as appropriate.

**Results:**

On average, recipients that reached gestation were significantly younger than the ones that did not. We also observed a significant positive effect of constant dose estrogen therapy on pregnancies.

**Conclusions:**

Patient age and response to estradiol therapy are important factors in the attainment of the best possible outcomes in cycles with donor oocytes.

## INTRODUCTION

Cycles with donor oocytes were first performed in the 1980, as a treatment for premature ovarian insufficiency (POI). In vitro fertilization (IVF) with donor oocytes has gained more acceptance for the treatment of age-related diminished ovarian reserve (DOR).

The use of donor oocytes has been widely accepted around the world. According to the Human Fertilization and Embryology Authority (HFEA), UK’s independent fertility treatment regulator, 3,924 women underwent in vitro fertilization cycles with donor oocytes in 2016, a 105% increase in relation to 2006 (HFEA, 2019). Data from the Centers for Disease Control and Prevention (CDC), which uses the United States’ National Surveillance System for Assisted Reproduction as a source, indicate that 24,049 cycles of in vitro fertilization with donor oocytes were performed in 2018 (CDC, 2021).

The main indication for the use of donor oocytes is POI, characterized by menopause before the age of 40 years (Asch *et al*., 1987; Sills *et al*., 2010; Sinha & Kuruba, 2007). Genetic predisposition, autoimmune and enzymatic diseases, and infections are the most common conditions that lead to early menopause (Jankowska, 2017). In addition, cycles with donor oocytes may be recommended when there is a chance of passing a genetic condition to the offispring or motherhood must be postponed for social or professional reasons (Alvarenga *et al*., 2018).

Today, donor oocyte cycles represent the most successful assisted reproduction technology (ART) treatment worldwide. Pregnancy rates resulting from donor oocyte cycles continue to rise, currently with a live birth rate of about 50%, making it the most successful ART procedure (European IVF-monitoring Consortium, 2017).

Beyond the undisputable beneficial effects for women unable to use their own oocytes to achieve gestation, the use of donor oocytes in ART treatments brings unique opportunities to study and understand factors affecting classical IVF cycles. In donor oocyte programs the oocyte source and the endometrium represent independent entities contributing to embryo implantation and gestation. In this sense, several studies have been carried out to understand the role that endometrial, embryonic and/or hormonal factors play on IVF outcomes (Melnick & Rosenwacks, 2018). It has been acknowledged that in classical IVF treatment, when the patient uses her own oocytes, there are patient characteristics, as well as factors related to the cycle, that have an impact on live birth rates. These parameters include female age and weight, cause of infertility, treatment history, superovulation length, fertilization rate, embryo score, ovarian response, and endometrial thickness (Vaegter *et al*., 2017).

The present retrospective study describes the characteristics of donor oocyte cycles and analyzes the data relative to these cycles to determine the factors that might have affected clinical pregnancy and live birth outcomes in our assisted reproduction center.

## MATERIALS AND METHODS

### General considerations

The study was conducted in compliance with Resolution No. 466/12 of the Ministry of Health (Brazil, 2012) and submitted for the appreciation of the Ethics Review Board for Research with Human Beings at the University of Vale do Rio dos Sinos (UNISINOS, Porto Alegre, Brazil) and given certificate no. 32894820.8.0000.5344. This single-center retrospective study was performed between January 2017 and January 2020 and included donor oocyte recipients that underwent embryo transfer cycles.

The study presented minimal risks related to the identification of participants based on the data extracted from their medical charts. The authors of the study pledged to maintain the confidentiality of patient identification information and ensuring patient anonymity. For data collection, a report was generated with information referring to the variables under study, and each patient was identified by a sequential number defined according to the date of completion of their donor oocyte cycles. A Data Use Disclaimer and a Data Lending Agreement was signed by the technical director of the institution where the study took place.

### Study population

The outcomes of 213 embryo transfers in donor oocyte cycles corresponding to 148 patients and their treatment characteristics were analyzed. Embryos were generated by intracytoplasmic sperm injection (ICSI) using fresh or cryopreserved donor sperm. Age of the oocyte donors ranged from 25 to 30 years. Semen parameters were not taken into account in our analysis.

For purposes of analysis, egg recipients were split into three groups according to age (<34; 35-39; > 40 years) and particular attention was given to the putative effect of preimplantation genetic testing for aneuploidies (PGT-A) on recipient pregnancy and implantation rates. Additional analyses were performed by grouping cycles according to outcomes of clinical pregnancy (positive or negative) and live birth (positive or negative).

The parameters considered in statistical analysis were cycles with positive clinical pregnancy with the presence of a gestational sac, regardless of whether it ended in live birth or miscarriage, and cycles with negative clinical pregnancy (negative human chorionic gonadotropin – hCG – test) and biochemical pregnancy (positive hCG test without a gestational sac). Cycles ended with the delivery of a healthy baby were categorized as positive deliveries; other outcomes were considered as negative deliveries.

### Endometrial preparation protocols

Recipients with ovarian function and menses received depot gonadotropin-releasing hormone (GnRH) antagonist for pituitary desensitization. All women underwent endo-metrial stimulation with oral or vaginal estradiol (E2). Initial estrogen administration was performed at a constant dose (CD, 6mg/ day) for all patients. CD treatment was maintained until embryo transfer (ET) in women presenting a satisfactory response (growth) of the endometrial lining. Patients that did not respond to initial E2 stimulus received personalized, increasing dose (ID) E2 therapy. The ID of E2 was administered as follows: oral E2 started at 6mg/day from day 1 until the day of the first ultrasound scan. When endometrial growth was not satisfactory, 8mg/day of E2 was recommended. This E2 dose was maintained until ET. One patient used vaginal ID estrogen with a dose similar to the oral ID protocol.

E2 supplementation was offered for 17 to 21 days, depending on the day the embryos were available for transfer and whether endometrial growth was satisfactory. Progesterone supplementation was performed with 1 (400mg) or 2 (800mg) intravaginal capsules (Utrogestan) or intravaginal progesterone gel (Crinone), both administered every 12h from the day of embryo transfer until the 12^th^ week of gestation in case of a positive hCG test performed 14 days after embryo transfer. Minor variations in endometrial preparation protocol can occur based on medical preferences or patient needs.

Gamete manipulation, oocyte insemination, embryo culture, PGT-A biopsy and embryo cryopreservation were performed according to standard protocols used in the IVF laboratory of our assisted reproduction center.

### Embryo classification

The blastocysts were scored according to the Gardner and Schoolcraft grading system (Gardner *et al*., 2000), before biopsy in PGT-A cycles, before vitrification or transfer in non-PGT-A cycles. Inner cell mass (ICM) was rated as A, B or C based on whether they presented tightly packed cells, loosely packed cells, or just a few cells, respectively. Trophectoderm was scored as A, B or C, based on whether they appeared as many cells organized in a confluent epithelium, several cells organized in a loose epithelium or a few large cells, respectively. Embryos were categorized according to the combination of ICM and TE scores as: BL1 (AA, AB or BA), BL2 (BB or CB) or BL3 (BC or CC). Embryo classification was performed by a clinical embryologist and reviewed by a senior embryologist.

### Statistical analysis

Data were entered into Microsoft Excel and later exported to IBM SPSS version 20.0 for statistical analysis. Categorical variables were described by frequencies and percentages. The normality of the quantitative variables was evaluated using the Kolmogorov-Smirnov test. Quantitative variables with a normal distribution were described by mean values and standard deviation, while variables with an asymmetric distribution were described using median values and interquartile ranges (25^th^ and 75^th^ percen-tiles). To associate categorical variables, the Chi-squared test or the Chi-squared test with Yates correction in 2x2 tables was used. Quantitative variables with a normal distribution were compared between groups using Student’s t-test for independent samples and the ones with an asymmetric distribution were compared based on the Mann-Whitney test. Factors that showed significant associations in bivariate analysis for at least one of the outcomes were included in Poisson regressions with robust variance. A significance level of 5% was considered.

## RESULTS

Data from 213 donor oocyte cycles performed on 148 patients were collected. A total of 108 recipients (73%) were aged 40 years or older during IVF treatment and the most frequent diagnosis was diminished ovarian reserve (89.9%). For patients that presented more than one infertility factor, the main factor was considered for analysis. Table 1 shows the demographic characteristics of the included patients.

**Table 1:** Baseline data of included patients for a total of 213 donor oocyte cycles.

Variables	n=213
**Attempts, median (IR)**	1 (1-2)
**Donor egg recipient age, mean ± SD**	42.5±5.4
**Donor egg recipient age ranges, n (%)** ≤ 34 years 35-39 years ≥ 40 years	14 (6.6) 41 (19.2) 158 (74.2)
**Donor egg recipient BMI, median (IR)**	23.3 (21.6-25.7)
**Diagnosis of infertility, n (%)** Anatomic female factor Endocrine female factor Endometriosis Male factor Diminished ovarian reserve Others Unexplained	19 (8.9) 1 (0.5) 5 (2.3) 3 (1.4) 180 (84.5) 6 (2.8) 1 (0.5)

IR: interquartile range; SD: standard deviation; BMI: body mass index.

The number of mature donor oocytes used in each cycle varied from 6 to 10 and the rate of blastocyst formation per donor oocyte cycle was 66.3%. Table 2 shows additional data on treatment cycles and embryo transfers. Most of the donor oocytes were fresh (84.5%) and 84.5% of the cycles were performed due to diminished ovarian function. The vast majority of the women were on hormone replacement therapy (97.1%) for endometrial preparation. The median endometrial thickness at transfer was 8 mm after a median 19 days of E2 administration. Nearly half of the patients were on constant dose oral estrogen; the other half had E2 administration tailored according to endometrial response. Luteal phase support was predominantly performed with vaginal progesterone. Embryo transfer was performed mainly at the blastocyst stage. In 19.2% of the cycles the embryos underwent biopsy for genetic analysis. Most patients (67.6%) received one blastocyst scored as BL1 at transfer.

**Table 2:** Donor oocyte cycles and embryo transfer characteristics.

Variables	n=213
**Oocyte source, n (%)** Cryopreserved Fresh Fresh+ Cryopreserved	32 (15.0) 180 (84.5) 1 (0.5)
**GnRH (antagonist), n (%)**	42 (19.8)
**Endometrial preparation, n (%)** Hormone replacement cycle Spontaneous cycle monitoring	204 (97.1) 6 (2.9)
**Endometrial thickness, median (IR)**	8 (7-9)
**Estrogen replacement, n (%)** Oral constant dose Oral increasing dose Vaginal increasing dose	120 (58.3) 85 (41.3) 1 (0.5)
**Days on medication, median (IR)**	19 (17-21)
**Luteal phase, n (%)** Vaginal Combined None Not available	203 (96.2) 4 (1.9) 1 (0.5) 3 (1.4)
**Embryo cryopreservation stage, n (%)** Blastocyst Cleaving Embryo Combined	198 (93.0) 11 (5.2) 4 (1.9)
**Genetic diagnosis, n (%)** PGT-A None	41 (19.2) 172 (80.8)
**Number of embryos transferred, n (%)** 1 2	156 (73.2) 57 (26.8)
**Classification of the best embryo, n (%)** BL1 BL2 BL3 Cleaving embryos Morula	144 (67.6) 55 (25.8) 2 (0.9) 8 (4.8) 1 (0.5)

IR: interquartile range, PGT-A: preimplantation genetic testing for aneuploidies.

In the 213 cycles analyzed, the overall clinical pregnancy rate was 47.6% (n=101) and the live birth rate was 41.3% (n=88). Table 3 shows the results of the analysis of the putative effects of age and genetic testing on recipient pregnancy and live birth rates. No statistically significant differences were found between age groups or embryo tested or not with PGT-A.

**Table 3:** Association between donor egg recipient age and genetic testing with establishment of clinical pregnancy or live birth.

	n	Clinical pregnancy n (%)	*p* value	Live birth n (%)	*p* value
**Age range** ≤ 34 years 35 - 39 years ≥ 40 years	14 41 158	8 (57.1) 20 (48.8) 73 (46.5)	0.737	8 (57.1) 15 (36.6) 65 (41.1)	0.401
**Genetic diagnosis** PGT-A None	41 172	19 (46.3) 82 (48.0)	0.991	15 (36.6) 73 (42.4)	0.612

PGT-A: preimplantation genetic testing for aneuploidies.

However, when pregnant and non-pregnant patients were compared for age, statistical analysis revealed that pregnant patients were younger than non-pregnant ones (41.7 *vs*. 45.3 years; *p*=0.036). There was also a signif-cantly higher rate of live birth among women who underwent more than one transfer cycle and a significant increase in the number of clinical pregnancies in patients given constant dose oral E2. None of the other parameters showed a significant impact on clinical pregnancy or live birth rates.

Donor oocyte recipient age and E2 administration protocol had each a significant impact on pregnancy and live birth rates. The relationships between age and E2 administration and clinical pregnancy and live birth were further investigated using the robust Poisson regression test. Results showed a strong correlation between the administration of continuous E2 and clinical pregnancy (Table 5).

**Table 5:** Poisson Regression with robust variance of associated factors and infant birth and clinical pregnancy.

	Clinical pregnancy		Live birth	
	RR* (95%CI)	p	RR* (95%CI)	p
**Estrogen use** Continuous oral doses Increasing oral doses	1.55 (1.12-2.14) 1 (Ref.)	**0.009**	1.32 (0.94-1.87) 1 (Ref.)	**0.0114**

RR: relative risk; 95% CI: 95% confidence interval; Ref: reference category; *Adjusted to age and attempt number.

## DISCUSSION

The purpose of the present single-center study was to characterize donor oocyte cycles in relation to clinical pregnancy and live birth in patients seen from 2017 to 2020.

Almost three quarters (73%) of the donor egg recipients were aged 40 years or older. This finding agrees with previous reports (Lopes *et al*., 2015) and stresses the fact that, in recent decades, the majority of the patients who seek donor oocytes are aged 40 years or older.

Recipient age seems to have an important effect on donor oocyte cycle outcomes. We observed that, on average, the patients that reached clinical pregnancy were statistically younger than the patients unable to reach clinical pregnancy. In autologous IVF cycles, the impact of patient age on oocyte quality and cycle outcome is well established. One might expect that in donor oocyte cycles the effects derived from the age of the recipient would be related to uterine factors that affect embryo implantation and development. Endometrial receptivity, together with embryo quality, are key factors for implantation and full-term gestation to occur. It is known that in autologous IVF cycles, even when endometrial thickness is appropriate and embryo quality is high, treatment may fail, particularly when maternal age is high. Early surveys on donor oocyte cycles reported that age is deleterious to implantation and increases miscarriage rates (Moomjy *et al*., 1999; Toner *et al*., 2002; Yeh *et al*., 2014). Clinical pregnancy and live birth rates remain stable among patients aged between 25 and late 40s and decline markedly after the age of 50 years (Soares *et al*., 2005). The increased rate of miscarriage and decline in implantation rates in older patients suggest that physiological endometrial alterations occur with aging. These changes may be related to endometrial steroid receptors, altered blood flow, stromal angiosclerosis and other processes described in other animal species (Melnick & Rosenwacks, 2018). A recent study showed that gene expression of proteins related to infammation and stemness is significantly higher in endometrial samples of patients in their 40s when compared with women in their 20s (Kawamura *et al*., 2021). Thus, these genes may be used as markers for endometrial aging and age-related infertility.

Regarding endometrial preparation, E2 constant dose seems to favor clinical pregnancy in donor oocyte cycles, though it does not affect live birth rates. A similar outcome was described in a previous report by Madero *et al*. (2016). The authors observed an increased rate of biochemical pregnancy after the use of E2 constant dose in donor oocyte cycles with fresh embryo transfers. However, the same study did not detect differences between CD and ID oral and transdermal E2 supplementation.

Premature ovarian insufficiency was the main reason (88.3%) for implanting donor oocytes in the patients included in this study. The European Society for Assisted Reproduction (European IVF-monitoring Consortium, 2017) and a recent CDC report (CDC, 2021) described premature ovarian insufficiency as the leading cause for using donor eggs. POI renders women subfertile, years or even decades prior to usual age of menopause. Donor oocytes are the only option for women in this condition to fulfill their desire to become pregnant. Presently, donor oocyte programs offer the highest efficacy in terms of pregnancy and live birth rates among assisted reproduction technologies. Pregnancy rates after one donor oocyte cycle are around 50%, and cumulative pregnancy rates after four cycles increase to 70-80% (Ameratunga *et al.*, 2009; Melnick & Rosenwacks, 2018).

In the present study, the source of oocytes, fresh or frozen, did not seem to exert a significant impact on pregnancy or live birth rates in donor oocyte cycles. This may have happened due to the smaller number of frozen donor oocyte cycles, when compared with the number of fresh donor oocyte cycles. However, our results are in agreement with a recent report by Cornet-Bartolomé *et al*. (2020), in which the authors reported similar success rates in terms of pregnancy and live birth rates after fresh and rewarmed donor oocyte cycles. The authors emphasized the role of well- established cryopreservation protocols and well-trained embryologists, to ensure oocyte viability post-rewarming. According to these authors, post-cryo-preservation oocyte survival, fertilization, embryo quality and pregnancy rates are affected by laboratory procedures, particularly during vitrification and rewarming. The ability to cryopreserve oocytes and the excellent survival rates post-rewarming have allowed clinicians more freedom in scheduling donor oocyte procedures, without the need of donor-recipient synchronization (Melnick & Rosenwacks, 2018).

Although questionable, the use of PGT-A in donor oocyte cycles is a common practice worldwide. PGT-A was applied in 19,2% of the cycles in the present study showing no positive effect on pregnancy and live birth rates, in agreement with previous reports (Barad *et al*., 2017; Stewart *et al*., 2019; Doyle *et al*., 2020). Preimplantation genetic diagnosis is considered a milestone in medical practice; however, it must be indicated and used in a judicious and individualized manner, since it is an invasive technique that may cause harm to embryos, change the embryonic microenvironment, and cause stress on cell structure and metabolism.

Our study did not take into account the possible effects of sperm parameters on the analyzed donor oocyte cycles. Although an early study (Girsh *et al*., 2008) demonstrated a decline in sperm quality with male aging and an effect on donor egg program outcomes, recent publications showed that poor semen quality was not associated with outcomes in donor oocyte cycles (Patounakis *et al*., 2015; Capelouto *et al*., 2018; Blázquez *et al*., 2016; Aghajanova *et al*., 2021). Thus, based on these last references, we did not assess the impact of semen quality in donor oocyte cycle outcomes.

Single embryo transfers were performed in the majority (73.2%) of the donor oocyte cycles analyzed in this study. Although the Resolution No. 2.168/2017 of the Federal Board of Medicine (CFM, 2017) allows the transfer of more than one embryo depending on the age of the recipient, nearly two decades of practice indicate that the transfer of more than one embryo may increase the chances of a multiple pregnancy. High success rates obtained after the transfer of a single embryo in donor oocyte cycles does not justify the transfer of more than one embryo or the risks of complications due to a multiple pregnancy, which include a fivefold increase in the chances of stillbirth; a seven-fold increase in the chances of neonatal death; extreme prematurity; premature rupture of membranes; development of pre-eclampsia; and gestational diabetes, among others (Soares *et al*., 2005).

The limitations of our study are related to the fact that we did not assess oocyte donor characteristics or semen quality in the donor oocyte cycles analyzed. There are controversies in literature on whether donor factors, such as BMI, age and lifestyle exert an effect on donor oocyte procedures and outcomes. A similar situation occurs when semen parameters are considered in this mode of infertility treatment. The two parameters shall be investigated in a future study, where specific donor factors, E2 administration protocols and semen quality represent the central point of our research.

## CONCLUSIONS

The present study describes the putative cycle parameters that contributed to successful donor oocyte cycle outcomes. The source of oocytes, fresh or cryopreserved, did not affect pregnancy or live birth rates. Donor egg recipient age seems to have an impact on pregnancy rate, an effect possibly related to the endometrial aging process. E2 CD administration may exert a positive effect on pregnancy rates. However, further studies are needed to explore this hypothesis. In view of the high implantation and pregnancy rates, the transfer of a single embryo is advised in this mode of infertility treatment. In addition to expanding the ability to treat infertility, donor oocytes represent a valuable tool to study isolated ovarian and endometrial factors and their impact on reproductive outcomes.

## Figures and Tables

**Table 4:** Donor oocyte cycle characteristics: comparison between groups negative and positive for clinical pregnancy and live birth.

	Clinical pregnancy			Live birth		
	Negative n=111	Positive n=102	p value	Negative n=125	Positive n=88	p value
Age	45.3±5.4	41.7±5.3	**0.036**	43.0±5.3	41.8±5.4	0.091
Body mass index	23.9±3.5	24.1±4.1	0.915	23.9±3.7	24.1±4.0	0.734
**Attempted cycles** First attempt (n) More than one attempt (n)	62 49	71 31	0.061	70 55	63 25	**0.030**
**Days on medication**	19.2±3.6	19.0±4.9	0.449	19.2±3.8	18.9±4.9	0.250
**Endometrium thickness**	7.9±1.4	8.1±1.6	0.242	7.9±1.5	8.1±1.5	0.124
Oocyte source Fresh (n) Frozen (n)	97 14	83 18	0.318	108 17	72 15	0.376
**Estrogen replacement** Oral constant dose (n) Oral crescent dose (n)	53 55	67 30	**0.007**	65 56	55 29	0.124
**GnRH administration (n)**	20	22	0.582	23	19	0.709
**Endometrial preparation** Hormone replacement (n) Spontaneous cycle (n)	108 2	96 4	0.426	121 2	83 4	0.235
**Embryo classification** BL1 (n) Others (n)	72 39	73 29	0.322	70 55	63 25	0.371

The values represent the mean values for the groups plus or minus a standard deviation or number of cycles. Statistically significant differences are highlighted in bold type followed by the calculated *p* values.
